# Exercise-induced effects on asprosin and indices of atherogenicity and insulin resistance in males with metabolic syndrome: a randomized controlled trial

**DOI:** 10.1038/s41598-024-51473-1

**Published:** 2024-01-10

**Authors:** Agnieszka Suder, Karol Makiel, Aneta Targosz, Marcin Maciejczyk, Piotr Kosowski, Alon Haim

**Affiliations:** 1 Department of Anatomy, Faculty of Physical Rehabilitation, University of Physical Education, 31-571 Cracow, Poland; 2 Department of Physiology, Faculty of Medicine, Jagiellonian University Medical College, 31-531 Cracow, Poland; 3 Department of Physiology and Biochemistry, Faculty of Physical Education and Sport, University of Physical Education, 31-571 Cracow, Poland; 4 Department of Petroleum Engineering, Faculty of Drilling, Oil and Gas, AGH University of Science and Technology in Cracow, 30-059 Cracow, Poland; 5 Department of Endocrinology and Diabetes, Faculty of Health Sciences, Ben-Gurion University of the Negev, 653 Beer-Sheva, Israel; 6 Soroka University Medical Center, 151 Beer-Sheva, Israel

**Keywords:** Metabolic disorders, Metabolic syndrome, Obesity, Lifestyle modification, Weight management, Rehabilitation

## Abstract

Metabolic syndrome (MetS) development is associated with insulin resistance and obesity, with the progression of visceral adipose tissue playing a crucial role. Excessive adipose tissue is accompanied by an increase in the asprosin (ASP), which is responsible for carbohydrate metabolism and the regulation of hunger and satiety. Exercise affects the release of ASP, which may regulate metabolism accordingly. Due to the inconclusive results of the effect of exercise on ASP concentration in men with MetS, 12-week interventions were carried out in the following groups: EG1—aerobic training (n = 21, age: 34.21 ± 6.06, WC; waist circumference: 114.7 ± 10.93) and EG2—a combination of aerobic and resistance training (n = 21, age: 37.37 ± 7.08, WC: 114.8 ± 11.64) and compared with a control group (CG) of men with MetS without any intervention (n = 20, age: 38.26 ± 7.43, WC: 115.3 ± 10.54). Body composition, indicators of carbohydrate-lipid metabolism, and ASP were assessed four times: before the intervention, at 6 and 12 weeks of training, and 4 weeks after the training sessions. A comparison of the intervention influence on changes in the analyzed variables between the groups was performed using ANOVA test for dependent groups with post-hoc comparison. The effect size (ES) was also assessed using squared eta (η^2^). The implementation of aerobic training resulted in a decrease in ASP concentration (*p* = 0.03) within 6 weeks of the intervention, while in the CG a gradual increase in ASP was confirmed (*p* < 0.001). Aerobic-resistance training did not induce significant changes in ASP concentration but resulted in an increase in fat-free mass/fat mass (FFM/FM) ratio (*p* < 0.001), and a decrease (*p* = 0.04) in Homeostasis Model Assessment of Insulin Resistance (HOMA-IR). Changes in the visceral adipose tissue level indicate a gradual decrease in both the EG1 (*p* = 0.01) and EG2 (*p* = 0.04) groups. Both aerobic and aerobic-resistance exercises may have a regulatory effect, mainly by reducing visceral adipose tissue, on the improvement of metabolic disorders.

## Introduction

In recent years, considerable attention has been devoted to metabolic syndrome (MetS) due to its role in the development of diseases such as obesity, diabetes, and cardiovascular diseases (CVD). The pathogenesis of MetS is complex and multifactorial, and the mechanisms underlying its development and progression are not fully understood^1^. MetS encompasses a combination of cardiovascular and metabolic risk factors, including abdominal obesity, insulin resistance, glucose intolerance, dyslipidemia, non-alcoholic fatty liver (NAFL), and arterial hypertension^2,3^. The most commonly implicated factor in the development of MetS is visceral obesity and the resultant insulin resistance. Excessive expansion of visceral adipose tissue leads to the development of a low-grade inflammatory state and increased secretion of proinflammatory adipokines, disrupting proper insulin signaling and contributing to the etiopathogenesis of MetS^4,5^. The development of visceral fat is also associated with hyperinsulinemia, increased free fatty acids, elevated blood pressure, and decreased HDL-C, predisposing individuals to thrombosis and other cardiovascular incidents^6,7^. MetS involves multiple biological systems and leads to high morbidity and mortality due to cardiovascular and metabolic complications. It poses a significant challenge to public health, and its prevalence is increasing significantly worldwide^6,8,9^. The increase in the risk of developing MetS is influenced by the patient's lifestyle, the presence of obesity in the family, genetic predispositions, and environmental factors, including the place of residence^10,11^. Analysis of populations with varying degrees of cardiovascular risk confirmed an increased risk of cardiovascular incidents with the rising number of components of MetS^12^. According to current findings, novel markers of atherogenicity and surrogate markers of insulin resistance, such as CRI-II, may be useful in predicting the risk of heart diseases^13,14^.

One protein that can significantly impact the development and progression of MetS and CVD is asprosin (ASP)^15,16^. ASP, named after the Greek word for "white", is synthesized in white adipose tissue^16^. It is responsible for glucose homeostasis, influences insulin metabolism, and the nutritional status of the body. Injection of ASP in mice leads to increased glucose release from the liver. Serum ASP levels are pathologically elevated in individuals with insulin resistance^16^. Patients with newly diagnosed type 2 diabetes and prediabetes exhibit significantly higher fasting serum ASP levels compared to the control group^17,18^. Similar situations are observed in mouse models of insulin resistance (such as the Ob mutation and diet-induced obesity). Reduction of ASP protects against hyperinsulinemia associated with MetS. ASP affects the nutritional state of the body by regulating feelings of hunger and satiety^16^. It has been demonstrated that ASP molecules cross the blood-brain barrier and directly stimulate orexigenic AgRP+ (agouti-related protein) neurons, activating the appetite center in the hypothalamus^19^.

Engaging in regular exercises enhances the outcomes of metabolic disorders by facilitating the redistribution of energy substrates, promoting the reduction of fat mass, and mitigating inflammatory processes^20^. Aerobic training leads to a significant increase in energy expenditure and creates advantageous conditions to decrease excessive adipose tissue mass, whereas resistance training is of significant importance to increasing fat free body mass, which results in higher insulin sensitivity and efficiency in maintaining and increasing the resting metabolic rate^21^. The results of studies on the effect of physical exercise on ASP concentration remains a relatively underexplored area^22^. In studies involving both men and women with normal body weight, as well as obese women, Schumann et al.^23^ observed no changes in ASP levels following a single bout of aerobic exercise. Conversely, in young women subjected to a single session of anaerobic exercise, Więcek et al.^24^ reported an elevation in serum ASP levels. In a study by Zarei et al., resistance training, aerobic training, and high intensity interval training (HIIT) led to decreased ASP and glucose levels after 8 weeks of intervention in a group of young men^25^. The application of moderate-intensity aerobic exercises may also lead to a decrease in serum ASP levels in overweight and obese individuals^26^. In the study by Jahangiri et al.^27^, it was demonstrated that engaging in various forms of resistance exercises for 12 weeks in three intervention groups resulted in a significant reduction in serum ASP levels and adipose tissue levels in obese men across all three groups. The authors attribute the observed disparities in exercise-induced changes in ASP concentrations to variations in the intensity and nature of the applied interventions^27^. The application of a combination of aerobic exercise with a low-energy diet also leads to a significant decrease in ASP, as well as correlated indicators of insulin resistance (HOMA-IR), adipokine levels such as TNF-alpha, IL-6, and adipose tissue levels^28^. Further investigations of a more comprehensive and extended nature are imperative to deepen our understanding of this intricate relationship. In our project, we hypothesized that aerobic resistance training would be associated with more favorable changes in body composition, such as an increase in fat-free mass to fat mass (FFM/FM) ratio and a reduction in visceral fat, and would consequently result in greater reductions in ASP concentrations, atherogenicity and insulin resistance rates compared to aerobic training in men with MetS.

## Results

In the intervention groups: EG1—performing aerobic exercises, and EG2—performing a combination of aerobic-resistance exercises, several favorable changes in the body composition of men with MetS were observed (Table 1). In EG1, a significant decrease (0.65%) in BMI (*p* = 0.01) between measurements was confirmed, but no significant changes were observed in the EG2 and CG groups.

**Table 1:** Body composition: body mass index (BMI), fat free mass to fat mass ratio (FFM/FM ratio) and android body fat (ANDR) in the aerobic group (EG1), aerobic-resistance group (EG2), and control group (CG).

	Group	Week 1, baseline	Week 6, intervention	Week 12, intervention	Week 16, follow up	Between measurements comparison *p*-value and ES			
		***X*®** ± SD	***X*®** ± SD	***X*®** ± SD	***X*®** ± SD	Test ANOVA (ES)	d 6–1 (ES)	d 12–1 (ES)	d 16–1 (ES)
BMI	EG1	34.57 ± 4.58	33.80 ± 4.59	33.87 ± 4.79	33.92 ± 4.94	0.01 (0.01)	< 0.001 (1.58)	0.01 (0.89)	0.01 (0.74)
	EG2	33.14 ± 4.32	33.12 ± 4.03	32.62 ± 4.24	29.98 ± 10.43	0.31 (0.04)	0.72 (0.11)	0.17 (0.42)	0.31 (0.31)
	CG	33.20 ± 4.31	33.72 ± 4.48	34.02 ± 4.56	34.15 ± 4.81	0.24 (0.00)	0.65 (0.13)	0.13 (0.48)	0.21 (0.39)
	*p*-value between groups	0.62	0.89	0.67	0.27				
FFM/FM ratio	EG1	1.75 ± 0.36	1.81 ± 0.41	1.82 ± 0.44	1.85 ± 0.41	0.04 (0.01)	0.05 (0.59)	0.04 (0.66)	0.03 (0.67)
	EG2	1.72 ± 0.31	1.84 ± 0.35	1.88 ± 0.33	1.92 ± 0.33	< 0.001 (0.04)	< 0.001 (1.13)	< 0.001 (1.30)	< 0.001 (1.71)
	CG	1.77 ± 0.36	1.73 ± 0.38	1.70 ± 0.35	1.64 ± 0.36	0.21 (0.01)	0.38 (0.33)	0.64 (0.16)	0.26 (0.40)
	*p-*value between groups	0.91	0.69	0.44	0.17				
ANDR (kg)	EG1	21.14 ± 7.92	19.94 ± 7.81	19.75 ± 7.88	19.43 ± 7.43	0.01 (0.01)	0.01 (0.84)	0.01 (0.85)	0.01 (0.90)
	EG2	19.18 ± 7.66	18.08 ± 6.99	17.07 ± 6.87	17.42 ± 7.31	0.04 (0.01)	0.11 (0.50)	0.02 (0.75)	0.09 (0.54)
	CG	19.79 ± 7.55	20.74 ± 8.59	21.89 ± 9.37	22.69 ± 8.95	0.95 (0.00)	0.81 (0.08)	0.55 (0.20)	0.89 (0.05)
	*p-*value between groups	0.78	0.63	0.30	0.27				

d 6–1, d 12–1, d 16–1 – differences in results obtained after 6 and 12 weeks of interventions, respectively, and after 4 weeks of follow-up in relation to measurements taken before interventions, ***X*®** mean, *SD* standard deviation, p < 0.05 – statistically significant difference, *ES* effect size.

In the EG1, an increase in the FFM/FAT ratio (*p* = 0.04) was demonstrated (Table 1). The progressive recomposition in favor of FFM reached significance after 12 weeks of intervention and was also confirmed after the observation period (increase 6%, *p* = 0.03). In the EG2, a change in the proportion between FFM and FAT was also confirmed (*p* < 0.001). The highly probable and substantial effect size of 1.71 after 16 weeks indicates the magnitude of the observed phenomenon. However, no changes in the FFM/FAT ratio were observed in the CG group.

Changes in the visceral adipose tissue level (ADNR) indicate a gradual decrease (respectively 8% and 9%) between measurements in both the EG1 (*p* = 0.01) and EG2 (*p* = 0.04) groups, while no changes were observed in the CG (Table 1).

Analyzing insulin resistance indices, it was demonstrated that after aerobic training (EG1), no changes were found in the level of HOMA-IR (Table 2). After the applied aerobic-resistance training (EG2), an initial increase followed by a 42% decrease in HOMA-IR between measurements (*p* = 0.04) during the follow-up period were observed. In the CG, no changes were found in the level of HOMA-IR. A difference between the EG2 and CG was confirmed after 12 weeks of the study (*p* = 0.04).

**Table 2:** Concentrations of homeostatic model assessment (HOMA-IR)—Eq. (1), triglycerides (TG) and Castelli’s risk index II (CRI II)—Eq. (3) in the participants’ blood in the aerobic group (EG1), aerobic-resistance group (EG2), and control group (CG).

	Group	Week 1, baseline	Week 6, intervention	Week 12, intervention	Week 16, follow up	Between measurements comparison *p*-value and ES			
		***X*®** ± SD	***X*®** ± SD	***X*®** ± SD	***X*®** ± SD	Test ANOVA (ES)	d 6–1 (ES)	d 12–1 (ES)	d 16–1 (ES)
HOMA-IR	EG1	4.53 ± 3.51	3.88 ± 4.66	4.31 ± 3.57	3.58 ± 3.45	0.29 (0.01)	0.39 (0.29)	0.10 (0.60)	0.09 (0.60)
	EG2	3.91 ± 2.08	4.91 ± 3.16	2.73 ± 1.28	2.26 ± 0.90	0.03 (0.14)	0.06 (0.67)	0.28 (0.37)	0.04 (0.77)
	CG	6.03 ± 5.05	5.26 ± 2.09	6.18 ± 4.77	5.60 ± 5.82	0.79 (0.01)	0.81 (0.08)	0.31 (0.36)	0.97 (0.01)
	*p-*value between groups	0.28	0.57	0.04*	0.15				
TG (mmol/L)	EG1	1.66 ± 0.81	1.60 ± 0.87	1.68 ± 0.76	1.50 ± 0.66	0.38 (0.01)	0.80 (0.08)	0.21 (0.43)	0.39 (0.29)
	EG2	1.90 ± 1.22	1.96 ± 1.15	1.80 ± 0.67	1.71 ± 0.62	0.25 (0.04)	0.11 (0.56)	0.13 (0.53)	0.57 (0.19)
	CG	2.47 ± 1.91	2.74 ± 2.83	3.56 ± 4.13	2.39 ± 2.07	0.14 (0.04)	0.06 (0.72)	0.21 (0.46)	0.08 (0.66)
	*p-*value between groups	0.29	0.28	0.11	0.22				
CRI II	EG1	2.80 ± 1.38	2.51 ± 0.98	2.40 ± 1.31	2.78 ± 1.24	0.02 (0.02)	0.99 (0.00)	0.01 (0.97)	0.95 (0.02)
	EG2	3.04 ± 0.91	2.73 ± 0.78	2.79 ± 0.83	2.92 ± 0.83	0.08 (0.03)	0.04 (0.73)	0.10 (0.58)	0.14 (0.51)
	CG	3.23 ± 0.83	3.18 ± 1.15	2.78 ± 0.57	3.24 ± 1.06	0.69 (0.02)	0.18 (0.58)	0.38 (0.36)	0.46 (0.30)
	*p-*value between groups	0.58	0.32	0.53	0.57				

*Post-hoc: EG2-CG, p = 0.04; d 6–1, d 12–1, d 16–1 – differences in results obtained after 6 and 12 weeks of interventions, respectively, and after 4 weeks of follow-up in relation to measurements taken before interventions, ***X*®** mean, *SD* standard deviation, p < 0.05 – statistically significant difference, *ES* effect size.

The analysis of atherogenicity indices revealed that after aerobic training (EG1), a 14% decrease in the CRI II index (*p* = 0.01) was confirmed after 12 weeks of intervention (Table 2). The level of triglycerides (TG) did not undergo significant changes during the analyzed period in the examined group. The application of aerobic-resistance training (EG2) led to a decrease in the CRI II level (*p* = 0.04) after 6 weeks of intervention, while other parameters of the lipid profile did not undergo significant changes. In the CG, no changes were found in the level of CRI II and TG (Table 2).

**Figure 1:**
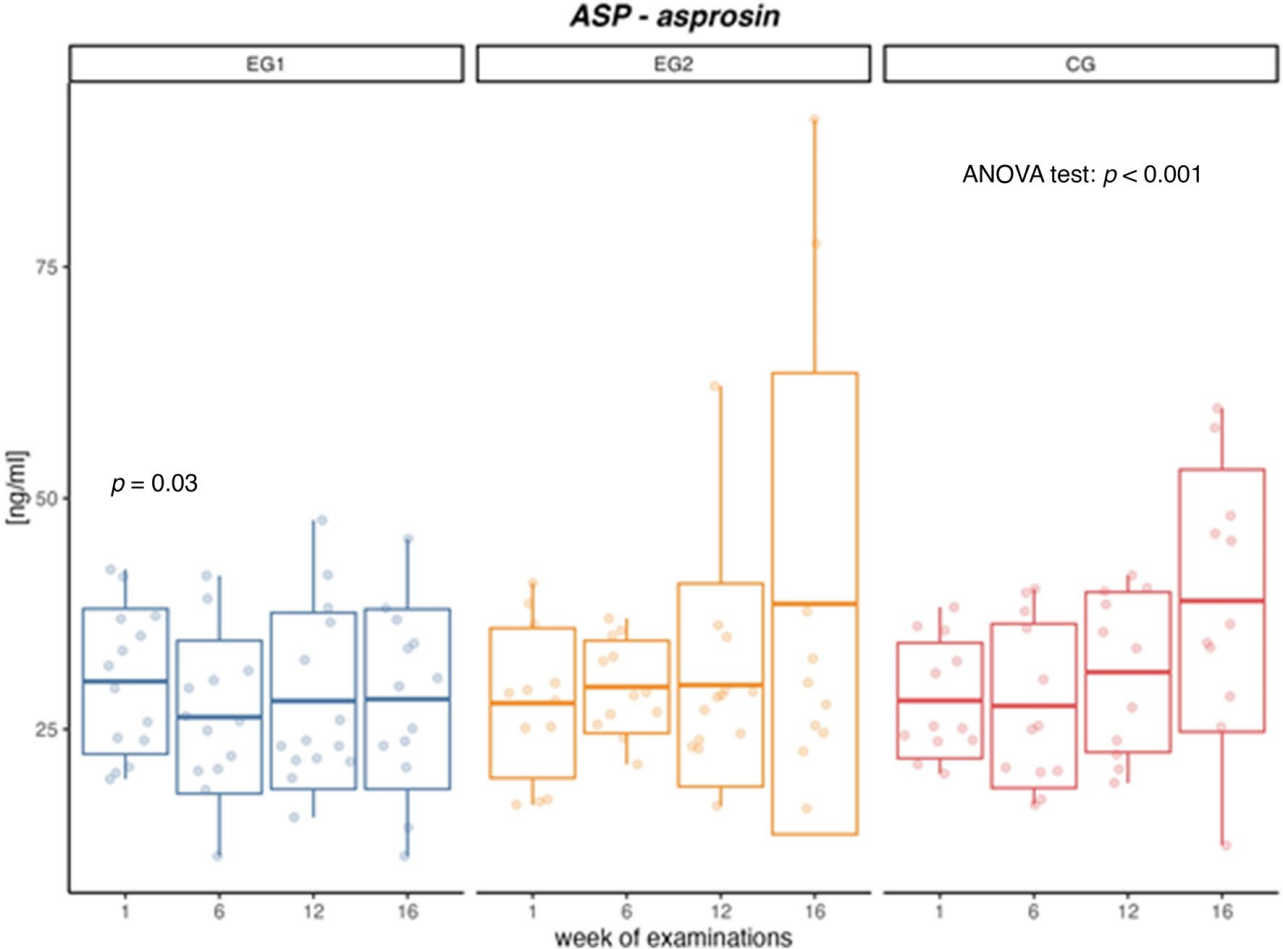
Changes in asprosin (ASP) concentration [ng/ml] in aerobic group (EG1), aerobic-resistance group (EG2) and control group (CG) during weeks of examinations.

After implementing a six-week aerobic training intervention in the examined men (EG1), a 13% decrease in ASP concentration (*p* = 0.03) was confirmed (Table 3, Fig. 1). After a four-week follow-up period, the ASP concentration remained slightly lower than the baseline level; however, the required level of significance was not achieved. After the application of aerobic-resistance training intervention (EG2), no significant changes were found in the concentration of ASP, both after six and twelve weeks of intervention, as well as during the follow-up period. In the CG, an increase in ASP concentration (*p* < 0.001) was confirmed between the measurements and after the sixteen-week observation period (*p* = 0.01) (Table 3, Fig. 1).

**Table 3:** Concentrations of asprosin (ASP) in participants’ blood plasma in the aerobic group (EG1), aerobic-resistance group (EG2), and control group (CG).

	Group	Week 1, baseline	Week 6, intervention	Week 12, intervention	Week 16, follow up	Between measurements comparison *p*-value and ES			
		***X*®** ± SD	***X*®** ± SD	***X*®** ± SD	***X*®** ± SD	Test ANOVA (ES)	d 6–1 (ES)	d 12–1 (ES)	d 16–1 (ES)
ASP (ng/ml)	EG1	30.18 ± 7.86	26.32 ± 8.28	28.07 ± 9.54	28.26 ± 9.73	0.15 (0.03)	0.03 (0.68)	0.37(0.26)	0.13 (0.45)
	EG2	27.83 ± 8.11	29.59 ± 5.00	29.78 ± 10.98	38.57 ± 24.94	0.33 (0.08)	0.84 (0.07)	0.77 (0.10)	0.38 (0.31)
	CG	28.10 ± 6.25	27.53 ± 8.88	31.18 ± 8.7	38.90 ± 14.17	< 0.001 (0.34)	0.78 (0.10)	0.76 (0.12)	0.01 (1.35)
	*p-*value between groups	0.68	0.56	0.73	0.22				

d 6–1, d 12–1, d 16–1 – differences in results obtained after 6 and 12 weeks of interventions, respectively, and after 4 weeks of follow-up in relation to measurements taken before interventions, ***X*®** mean, *SD* standard deviation, p < 0.05 – statistically significant difference, *ES* effect size.

In the intervention groups, no significant correlations were confirmed between ASP and the other variables (Table 4). Significant correlations were observed in the CG between ASP and TG levels. The remaining correlation dependencies are presented in Table 4.

**Table 4:** The value of the Pearson correlation for variables in the aerobic group (EG1), aerobic-resistance group (EG2), and control group (CG).

	BMI	FFM/FM ratio	ANDR	HOMA-IR	TG	CRI II	ASP
ASP EG1 [ng/ml]	0.02	0.05	− 0.13	− 0.02	0.16	0.17	1.00
ASP EG2 [ng/ml]	0.15	− 0.23	0.20	0.13	− 0.16	0.00	1.00
ASP CG [ng/ml]	− 0.13	0.11	− 0.10	0.07	− 0.36*	− 0.28	1.00
HOMA-IR EG1	0.54*	− 0.34*	0.49*	1.00	0.64*	0.24	− 0.02
HOMA-IR EG2	0.62*	− 0.34*	0.58*	1.00	− 0.20	− 0.14	0.13
HOMA-IR CG	0.19	0.38*	− 0.10	1.00	0.30	0.10	0.07
CRI II EG1	0.48*	− 0.55*	0.58*	0.24	0.49*	1.00	0.17
CRI II EG2	0.01	0.19	− 0.20	− 0.14	0.20	1.00	0.00
CRI II CG	0.27	− 0.13	0.20	0.10	0.09	1.00	− 0.28

*Statistically significant value p < 0.05; ASP EG1—concentrations of asprosin in EG1 taken from the four timepoints; ASP EG2—concentrations of asprosin in EG2 taken from the four timepoints; ASP CG—concentrations of asprosin in CG taken from the four timepoints; HOMA-IR EG1—concentrations of homeostatic model assessment in EG1 taken from the four timepoints; HOMA-IR EG2—concentrations of homeostatic model assessment in EG2 taken from the four timepoints; HOMA-IR CG—concentrations of homeostatic model assessment CG taken from the four timepoints, CRI II EG1—concentrations of Castelli’s risk index II assessment in EG1 taken from the four timepoints; CRI II EG2 — concentrations of Castelli’s risk index II in EG2 taken from the four timepoints; CRI II CG—concentrations of Castelli’s risk index II CG taken from the four timepoints, FFM/FM ratio—fat free mass to fat mass ratio, *ANDR* android body fat, *BMI* body mass index, *HOMA-IR* concentrations of homeostatic model assessment, *TG* triglycerides, *CRI II* Castelli’s risk index II.

## Discussion

The aim of this study was to compare the 12-week impact of two types of physical training on the concentration of asprosin (ASP), as well as insulin resistance and atherogenicity indices in men with metabolic syndrome (MetS) compared to men with MetS who did not engage in physical activity. An assessment of changes in these parameters after 4 weeks of observation without planned training was also conducted. The implementation of aerobic training led to a decrease in ASP levels as early as 6 weeks of intervention. The introduction of aerobic-resistance training did not significantly affect its level, while a significant increase in ASP concentration was observed in the CG during the study. Both intervention groups demonstrated favorable changes in atherogenicity expressed by CRI II, and a decrease in insulin resistance was observed in the EG2, as indicated by the HOMA-IR index. Significant reductions in visceral fat and increases in the FFM/FM ratio were also confirmed in both intervention groups.

The demonstrated favorable changes (a 13 % decrease) in ASP levels, occurring in the group undergoing aerobic intervention after 6 weeks of training, may be attributed to a decrease in adipose tissue levels, as indicated by the increase in FFM/FM ratio, decrease in ANDR, and decrease in BMI during the same period. In studies examining long-term physical exercise interventions, Zarei et al.^25^ also reported a decrease in ASP concentrations in the intervention groups following the implementation of aerobic, resistance, and HIIT training conducted three times a week for eight weeks. A report on serum ASP concentrations during prolonged exercise confirmed that 8-week Nordic walking training at FAT max was associated with reduced blood ASP concentrations in young women with metabolic disorders^29^. Similarly, in a study of obese men leading a sedentary lifestyle, who underwent 12-week resistance training, a reduced concentration of ASP in serum was confirmed^27^. Similar beneficial results in reducing ASP levels were achieved by researchers using an 8-week swimming training program for rats with MetS^30^. Previous studies suggest differential exercise-induced changes in ASP concentration and the response may depend on gender, body fat levels and exercise metabolism (aerobic, anaerobic)^23,24,26^. Reductions in ASP concentrations were observed both after a single aerobic exercise^26^ and after several weeks of aerobic training at maximal fat oxidation^29^, but Więcek et al.^24^ reported also an increase ASP in young women after anaerobic exercise. Moreover, the greater reduction was observed in obese men in comparison to men with normal body composition^26^.

During the observation period without planned exercises in our study, we confirmed an increase in ASP concentration compared to the 12-week intervention, both in the intervention group (EG2) and the CG. Such fluctuation may be attributed to increased carbohydrate and fat consumption in the diet of the examined men, as demonstrated in our previous work^31^. The observed upward trend in circulating ASP concentrations in CG may corroborate reports associated with the risk of ASP accumulation in the bodies of individuals with MetS and the ensuing metabolic consequences. Elevated levels of ASP in the body induce increased appetite, leading to heightened energy intake. The accumulation of energy derived from dietary sources over an extended period results in an increase in adipose tissue mass. The augmented adipose tissue mass synthesizes additional amounts of ASP into the bloodstream, closing the cycle and further contributing to increased appetite and adipose tissue accumulation. Such a mechanism may contribute to the progression of obesity and its associated metabolic disorders^16,17^. In the CG, despite the lack of intervention, we confirmed an increase in fat consumption in the diet^31^. The disease of obesity and its comorbidity with MetS, without interventions aimed at improving health status, lead to progressive hormonal, metabolic, psychological, and numerous other disorders^32,33^.

Although ASP is primarily synthesized in adipose tissue, in our study, no correlations were confirmed between body composition indices and ASP. Other observations were made by Zhang et al.^17^ and Wang et al.^18^ in a group of individuals with prediabetes and patients with type 2 diabetes, where positive correlations were found between ASP levels and the HOMA-IR insulin resistance index. Positive correlations were also found for other factors, such as TG levels and fasting glucose.

Analyzing the HOMA-IR insulin resistance index, a 30 % decrease from the baseline value was confirmed after 12 weeks of aerobic-resistance intervention. The difference in HOMA-IR levels between EG2 and CG was 56 %. Changes in HOMA-IR levels in this type of training are mainly associated with changes in insulin concentration^34^. Processes related to changes in insulin resistance levels in the study group were also described in HOMA-AD and HOMA-TG indices, whose fluctuations closely reflect the obtained HOMA-IR values^31^.

In both intervention groups, changes in the FFM/FM ratio were confirmed. It is worth noting the scale and effects of the intervention observed in the group subjected to aerobic-resistance intervention. After 16 weeks, a 12 % increase in the ratio compared to the baseline measurement was confirmed. In the group performing aerobic training alone, the change was 6 %, which is half the amount. Moreover, the high level of significance in the changes within the EG2 group between individual measurement points and the high effect size confirm the impact of aerobic-resistance training on the recomposition of parameters, namely a significant increase in FFM and decrease in FM during the analyzed period. When engaging in aerobic training alone, favorable changes occur at a slower pace during the presented period. The observed phenomenon may be attributed to the significant influence of resistance training on muscle mass development, which is the main component of FFM in the EG2 group, as changes in fat tissue levels are similar in groups performing aerobic training alone and aerobic-resistance training^31^.

Due to the changes in the FFM/FM ratio observed in the aerobic-resistance group, no changes in BMI were confirmed. The development of muscle mass through aerobic-resistance training maintained body weight at a similar level, despite a reduction in fat tissue. On the other hand, in the group with aerobic intervention, a significant decrease in BMI between measurements reflects a decrease in body weight due to lost fat tissue, as demonstrated in previous paper^35^.

The obtained results indicate significant correlations between HOMA-IR and FFM/FM ratio, ANDR, and BMI in the intervention groups. The negative correlation between HOMA-IR and FFM/FM ratio in the intervention groups, as well as the positive correlation between HOMA-IR and ANDR in these groups, indicate the importance of the proportion between fat-free mass and adipose tissue, as well as the distribution of adipose tissue in the process of insulin resistance. As the FFM/FM ratio increases, the level of insulin resistance expressed by the HOMA-IR index decreases, while the development of ANDR leads to an increase in insulin resistance. Visceral fat is a strong, independent predictor of dyslipidemia^36,37^ and insulin resistance^38^, and changes in visceral fat tissue are associated with concomitant changes in glucose tolerance and insulin resistance^39^. Visceral fat is an independent factor in overall mortality in men^40^.

In the group with aerobic intervention, despite the lack of correlation between the indicator of atherogenicity (CRI II) and HOMA-IR, similar relationships between CRI II and body composition parameters were confirmed. The significant correlations between CRI II and FFM/FM ratio, ANDR, and BMI confirm the value of assessing such body composition indices in studies on patients with MetS, as well as in the process of treatment and monitoring the progress of applied therapy. In the study by Son and Park^41^, it was confirmed that resistance training prevents the development of MetS by reducing HOMA-IR, TG, LDL cholesterol, percentage of FM, and increasing FFM and HDL in obese women with MetS. The authors of the report indicate that resistance training can be an effective therapeutic intervention in combating components of MetS and may reduce the risk of developing CVD^41^.

The scientific study has encountered several limitations that need to be addressed. Firstly, despite the initial intentions to maintain the participants' current diet and rigorously control their food intake, there was an unintended increase in the amount of energy supplied through food. Additionally, it should be noted that during the control procedure, the VO_2_ max test was omitted.

## Conclusions

The implementation of aerobic training in the group of men with MetS resulted in a decrease in ASP concentration within 6 weeks of intervention. Individuals who did not engage in training interventions showed a gradual increase in blood ASP levels. Aerobic-resistance training did not induce significant changes in ASP levels, but it led to more favorable changes in body composition and HOMA-IR levels compared to aerobic training alone. The applied interventions lowered the level of CRI II in men with MetS. The level of visceral fat was closely associated with the level of insulin resistance expressed by HOMA-IR and the atherogenicity expressed by CRI II. Changes in the visceral adipose tissue level indicate a gradual decrease in both intervention groups. Both aerobic and aerobic-resistance exercises may have a regulatory effect, mainly by reducing visceral adipose tissue, on the improvement of metabolic disorders.

## Methods

The study was a prospective, randomized, controlled trial aimed at investigating the effects of two types of twelve-week physical training interventions (aerobic training vs. combined aerobic-resistance training) on body composition, ASP levels, and selected metabolic syndrome indicators in men with MetS compared to men with MetS who did not engage in any training (control group, CG). Participants from all three groups were observed for four weeks without any planned training sessions, which served as a follow-up period.

Due to the nature of the interventions, a blinded design was not implemented. However, the laboratory staff, biostatisticians, and analytical team were unaware of the participants' group assignments. The research project obtained approval from the Local Ethics Committee of the Regional Chamber of Physicians in Krakow (90/KBL/OK/2020) and all methods were performed in accordance with the guidelines of the Declaration of Helsinki. Informed consent was obtained from all subjects involved in the study. The study was registered in the Australian New Zealand Clinical Trials Registry under the registration number ACTRN12622001394730 (31/10/2022) and followed CONSORT guidelines. A detailed description of the research methods was presented in previous papers^31,34,35^.

*The inclusion criteria for the study were*: male gender, age between 30 and 45 years, medical clearance for engaging in aerobic-resistance training, written consent to voluntary participation in the research project, increased waist circumference ≥ 94 cm, and two of the following criteria: triglyceride levels >150 mg/dL (1.7 mmol/L) or treated hypertriglyceridemia, HDL-C levels <40 mg/dL (1.03 mmol/L) in men or lipid disorder treatment, systolic blood pressure (SBP) ≥130 mm Hg or diastolic blood pressure (DBP) ≥85 mm Hg or previously diagnosed hypertension treatment, fasting glucose level ≥100 mg/dL (5.6 mmol/L) or pharmacological treatment of type 2 diabetes (T2DM).

*Exclusion criteria included:* not meeting the inclusion criteria, unwillingness to continue with the interventions (more than 10% missed training sessions), ischemia, heart failure, arrhythmias, severe pulmonary hypertension (mean pulmonary artery pressure > 55 mm Hg), symptomatic aortic stenosis, acute myocarditis, pericarditis, uncontrolled blood pressure (> 180/110 mm Hg), aortic dissection, uncontrolled diabetes, psychiatric disorders during the study period, health problems (orthopedic, neurological) that hindered mobility, participation in other forms of physical activity during the project, lack of written consent to participate in the study.

The study included 62 men with a mean age of 37 ± 7 years who met the inclusion criteria. The participants were randomly assigned to three groups using simple randomization based on sealed opaque envelopes from a container (assignment concealment procedures):

1. Experimental group 1 (EG1) consisted of men with MetS (n = 21) performing aerobic exercises.
2. Experimental group 2 (EG2) consisted of men with MetS (n = 21) performing a combination of aerobic-resistance exercises.
3. Control group (CG) consisted of men with MetS (n = 20) who did not engage in any physical activity.

The basic characteristics of the research participants in the aerobic group (EG1), aerobic-resistance group (EG2), and control group (CG) are presented in Table 5. There were no differences between EG1, EG2, and CG with respect to age, number of parameters of MetS confirmed in the examined males, and basic anthropometric parameters before the interventions.

**Table 5:** Characteristics of the research participants in the aerobic group (EG1), aerobic-resistance group (EG2), and control group (CG).

Index	Group			*p*-value
	EG1	EG2	CG	
Age [years]	34.21 ± 6.06	37.37 ± 7.08	38.26 ± 7.43	0.20
MetS criterion acc. to IDF	3.07 ± 0.83	3.25 ± 0.86	3.47 ± 0.74	0.30
BF [%]	38.03 ± 4.82	37.33 ± 4.30	37.22 ± 4.37	0.87
FFM [%]	63.09 ± 4.81	62.56 ± 5.23	59.23 ± 16.97	0.58
WC [cm]	114.7 ± 10.93	114.8 ± 11.64	115.3 ± 10.54	0.93
WHTR	63.37 ± 6.22	63.90 ± 5.97	63.72 ± 4.99	0.82

*MetS* number of metabolic syndrome parameters that meet the criteria of recognition by IDF (International Diabetes Federation), *BF* percent of body fat, *FFM* percent of fat-free mass, *WC *waist circumference, *WHTR* waist-to-height ratio.

The assignment to the intervention was parallel. The implementation was conducted by a trainer. The subjects were recruited and underwent intervention from September 2020 to July 2021, ensuring that the study groups achieved the minimum required sample size. There were no occurrences of harm and adverse event reported during the period of trial. However not all participants completed the project according to the assumptions. The exclusion factors were: absence during control measurements—9 individuals, changes in diet (alcohol consumption)—2 individuals, infectious diseases—3 participants, low attendance (< 90%) during training—3 patients.

The primary outcome measures used in this study were an assessment of body composition and an asprosin concentration, and secondary outcome measures were an assessment of the levels of carbohydrate-lipid metabolism indicators. The following research methods were applied and conducted four times: before the start of training, during the project (after 6 weeks and 12 weeks of training), and 4 weeks after the completion of training sessions (follow-up):

1. *Body composition and anthropometric measurements*. Dual-energy X-ray absorptiometry (DEXA) was used to assess body composition, including fat mass content (FM) [kg], fat-free mass (FFM) [kg], android body fat (ANDR) [kg], and body mass index (BMI) [kg/m^2^]. The Lunar Prodigy Primo PR + 352163 device was used for the measurements. The FFM/FM ratio was calculated based on the obtained data. Additionally, body height (BH) [cm], body mass (BM) [kg], and waist circumference (WC) [cm] were measured. All measurements were taken in a standing position, in underwear, with an accuracy of 1 mm for BH and WC and 50 g for BM.
2. *Hormonal and biochemical blood indicators*. Fasting blood samples were collected in the morning, after a 24-h training break, from the median cubital or median vein into Vacumed® tubes (F.L. Medical, Torreglia, Italy) by an experienced nursing team. The collected blood was centrifuged (RCF 1000 × g) immediately after collection for 15 min at 4 °C (MPW-351R, MPW Med. Instruments, Warsaw, Poland), and the serum was collected and stored at -80 °C for further analysis (BIO Memory 690L, Froilabo, Paris, France).

The levels of human asprosin (ASP) were measured using commercially available ELISA kits following the manufacturer's protocol. The detection kits for asprosin (ELISA Kit catalog number 201-12-7691) were purchased from Shanghai Sunred Biological Technology Co. (Shanghai, China). An ELx 808 spectrophotometric microplate reader (BioTek, Winooski, Vermont, USA) was used to determine the optical density at 450 nm. The measurements were performed in the in the Laboratory of Genetics and Molecular Biology at the Department of Physiology, Jagiellonian University Medical College, Cracow, Poland.

The serum glucose level (GL) [mmol/L] was determined by an enzymatic method using the Cobas c701/702 biochemical analyzer. The serum insulin level [µIU/mL] was determined by electrochemiluminescence (ECLIA) using the Cobas e801 instrument. The measurements were carried out according to the manufacturer's recommendations using dedicated reagents for GLUC3 and Elecsys Insulin analyzers. The value of the HOMA-IR (Homeostatic Model Assessment of Insulin Resistance) index was calculated using the formula^42^:

1 $${\text{HOMA-IR }} = {\text{ fasting insulinemia }}\left( {{\text{mU}}/{\text{mL}}} \right) \, \times {\text{ fasting glycemia }}\left( {{\text{mmol}}/{\text{L}}} \right)/{22}.{5}$$

Total cholesterol (TC), triglycerides (TG), and high-density lipoprotein cholesterol (HDL-C) were determined using the spectrophotometric method with the clinical chemistry analyzer Architect ci-4100. The LDL-C fraction was calculated using the formula:

2 $${\text{LDL-C }}\left( {{\text{mmol}}/{\text{L}}} \right) \, = {\text{ TC }}\left( {{\text{mmol}}/{\text{L}}} \right) \, {-}{\text{ HDL}} - {\text{C }}{-} \, \left( {{\text{TG }}\left( {{\text{mmol}}/{\text{L}}} \right) \, /{ 2}.{2}} \right).$$

Castelli’s risk index-II (CRI II) was calculated according to the following formula^43^:

3 $${\text{CRI II}} = \, \left( {{\text{LDL}} - {\text{C }}\left( {{\text{mmol}}/{\text{L}}} \right) \, /{\text{ HDL}} - {\text{C }}\left( {{\text{mmol}}/{\text{L}}} \right)} \right).$$

3. *Assessment of energy expenditure and dietary energy intake*. In order to control energy expenditures, the International Physical Activity Questionnaire (IPAQ) was used to assess daily energy expenditures. Total energy expenditure (TEE) was calculated as the sum of non-exercise thermogenesis estimated using the IPAQ questionnaire and energy expenditures associated with the interventions implemented in EG1 and EG2 groups.

The subjects were asked not to change their diet during the project. To assess the energy intake [kcal/day] of the participants’ diets, patients kept food diaries that were continuously analyzed during group training sessions. During each checkpoint, a dietitian conducted a detailed dietary interview for the past 3 days using the dietary record method. The data obtained from the interview were analyzed using the DietaPro program (version 4.0, Institute of Food and Nutrition, Warsaw, Poland) to quantitatively assess dietary habits and monitor changes in the diet during the intervention. The energy value of the diet was assessed in kcal/week.

The exercise interventions took place at a fitness club in Cracow under the supervision of a personal trainer. All training sessions were conducted at the same time of day (evening; 18-21) by the same personal trainer, in a room with the same temperature (22 degrees Celsius) and humidity. Attendance checklists were used to monitor adherence to the interventions. Participants who did not meet at least 90% of the training requirements during the 12-week period were excluded from the observation and statistical analysis.

The planning and monitoring of aerobic training intensity and resistance training load were individually determined based on the guidelines of the American College of Sports Medicine^44^. Heart rate (HR) during exercise was monitored using the Polar M200 GPS Running Watch with a wrist heart rate monitor. One-repetition maximum (1RM) was determined prior to resistance training. The load and number of repetitions were recorded and calculated using the 1RM calculator, using the Brzycki formula^45^.

The course of the aerobic intervention (Supplementary Table S1). The training sessions took place three times a week in groups of no more than 5 individuals and began with a five-minute warm-up on a treadmill (Technogym New Excite Run Now 500, Cesena, Italy) at 50% of maximum heart rate (HR max). Participants then increased the intensity of their training to 70% HR max by increasing the speed or incline on the treadmill, resistance on stationary bicycles (Technogym Artis, Cesena, Italy), or range of motion or resistance on elliptical trainers (Precor EFX556i Elliptical, Woodinville, WA, USA). The aerobic exercises primarily involved brisk walking or jogging on the treadmill. In case of musculoskeletal discomfort, participants had the option to switch to a different exercise equipment. The training was continuous, maintaining a steady HR level, and lasted for 45 minutes. The main segment of the training regimen concluded with one minute of brisk walking, followed by an additional minute of leisurely ambulation. Following the cool-down phase, participants engaged in a nine-minute session of stretching, culminating the training with respiratory exercises lasting one minute.

In previous studies, we demonstrated that the applied intervention in the form of aerobic training in the EG1 group was associated with a significant increase in energy expenditure [kcal/day] in each week of measurements compared to the first week of the study^31^. The total energy expenditures in the EG1 group were 823.37 ± 175.76 kcal/day after 6 weeks and 835.18 ± 234.05 kcal/day after 12 weeks of intervention. During the observation period, participants in the EG1 group maintained a high level of physical activity, amounting to 838.00 ± 350.75 kcal/day, maintaining an energy expenditure level [kcal/day] similar to that during the intervention^34^.

The course of the aerobic-resistance intervention (Supplementary Table S2). The combined aerobic-resistance training took place three times a week in groups of no more than 5 individuals. The training began with a five-minute aerobic warm-up on a treadmill at an intensity of 50% HR max.

The initial resistance training comprised three complex exercises involving the whole body, such as one-arm dumbbell row, squats, and push-ups, with four sets and 120 s breaks between them. Due to the body's adaptation to training, in the second week of the intervention, the resistance training procedure was changed to push-pull and the training volume was changed to 3 sets of 6 exercises with 90 s breaks. After 3 weeks of intervention, the training was performed in 3 series of 9 exercises with 60 s breaks. The load was gradually increased from the first week, from 50 1RM to 70% 1RM in the second and the remaining 10 weeks of intervention.

After the resistance exercises, an aerobic training element followed: participants trained at an intensity of 50% HR max in the first week and 70% HR max from the second week of the intervention on a treadmill (Technogym New Excite Run Now 500, Cesena, Italy), stationary bike (Technogym Artis, Cesena, Italy), or elliptical trainer (Precor EFX556i Elliptical, Woodinville, WA, USA). To avoid overloading the lower limbs, participants could use these three devices alternately. The duration of the resistance training session was 30, 35, and 40 minutes, followed by 20, 15, and 10 minutes of aerobic training, respectively. The training regimen concluded with one minute of brisk walking, followed by an additional minute of leisurely ambulation. Following the cool-down phase, participants engaged in a four-minute session of stretching, culminating the training with respiratory exercises lasting one minute.

The application of aerobic-resistance training in the EG2 group was associated with an increase in energy expenditure [kcal/day] in each week of measurements compared to the first week of the study^31^. The total energy expenditures in the EG2 group were 735.17 ± 119.64 kcal/day after 6 weeks and 797.89 ± 383.25 kcal/day after 12 weeks of intervention. During the observation period, participants in the EG2 group maintained a high level of physical activity, amounting to 749.17 ± 430.71 kcal/day, maintaining an energy expenditure level [kcal/day] similar to that during the intervention^31^.

### Statistical analysis

The Shapiro-Wilk test was initially conducted for the analyzed variables to check the hypothesis whether the sample follows a normal distribution. Due to the normal distribution of the majority of variables, differences between the intervention groups and the control group were estimated using one-way analysis of variance (ANOVA) for independent groups. A comparison of the intervention influence on changes in the analyzed variables between the experimental group (EG) and control group (CG) was performed using ANOVA test for dependent groups with post-hoc comparison (Tukey test). The effect size (ES) was assessed for the ANOVA test using squared eta (η^2^):

4 $${\eta }^{2}=\frac{{SS}_{effect}}{{SS}_{total}},$$

where squared eta (η^2^) is the ratio of the sum of squares (SS) for the effect divided by the total sum of squares (SS). The interpretation of the ES was as follows: 0.01 ≤ 0.05 (low effect), 0.06 ≤ 0.13 (moderate effect) and ≥0.14 (high effect).

Pearson correlation coefficient (r) was calculated. The interpretation of the Pearson correlation in the range <0–1> was as follows: 0 ≤ r < 0.3, no or very weak correlation; 0.3 ≤ r < 0.5, moderate correlation; 0.5 ≤ r < 0.7, strong correlation; 0.7 ≤ r ≤ 1, very strong correlation.

The number of participants required to demonstrate statistical significance was based on previously published studies in the field. The probability of error (α) of 0.05, the power (1–β) of 0.80, and the mean effect size (d) of 0.8 were used to calculate the sample size. The analysis was carried out according to the originally assigned groups.

In all analyses, effects were considered significant if the probability value (p) was less than the adopted significance level α = 0.05 (p < 0.05). R programming language, RStudio IDE, and ggplot2 package were used for the calculations.

### Supplementary Information

Supplementary Information.

## Data Availability

The datasets used and/or analysed during the current study available from the corresponding author on reasonable request.
