# Supplementary material for: Exercise-induced effects on asprosin and indices of atherogenicity and insulin resistance in males with metabolic syndrome: a randomized controlled trial
Source: Sci Rep. 2024 Jan 10;14:985. doi: 10.1038/s41598-024-51473-1 (PMC10782011; doi:10.1038/s41598-024-51473-1)
Supplement: Supplementary file 1 — Supplementary Information. [file 41598_2024_51473_MOESM1_ESM.docx]

Table S1. A general plan for the aerobic program designed for a collective involved in aerobic exercises (EG1).

| **Stage** | **Activity** | **Details** |
| --- | --- | --- |
| Warm-up | Treadmill walk | 5 minutes at 50% HR max |
| Main Training | Aerobic exercise | 45 minutes at 70% HR max. Options to adjust intensity: speed/incline on treadmill, resistance on bike, range/motion on elliptical. Brisk walking or jogging on the treadmill is primary. Switch equipment if necessary. |
| Cool-down & Post-training | Brisk walking & Leisurely walk | 1 minute each, followed by stretching (9 minutes) and respiratory exercises (1 minute) |

HR max – maximal heart rate

Table S2. A general strategy for a combined aerobic and resistance training program intended for a group engaged in aerobic–resistance exercises (EG2).

|  | **Week 1**  **Intervention** | **Week 2**  **Intervention** | **Week 3<**  **Intervention** |
| --- | --- | --- | --- |
| Duration of aerobic training [min] | 20 | 15 | 10 |
| Intensity of aerobic  [HR max] | 50 | 70 | 70 |
| Duration of resistance training [min] | 30 | 35 | 40 |
| Intensity of resistance training [% 1 RM] | 50 | 70 | 70 |
| Volume of resistance training [exercises x series x repetitions] | 3 x 4 x 15 | 6 x 3 x 12 | 9 x 3 x 12 |
| Breaks between series [min] | 2 | 1.5 | 1 |

HR max—maximal heart rate, 1RM—one repetition maximum.

Initially, the training regimen consisted of 3 comprehensive exercises engaging the entire body (FBW—full body workout), in the following sequence: bent isolated one-arm dumbbell row, push-ups with adjustable arm prop height, squats, 4 sets with 120-second breaks between them. This exercise sequence facilitated the activation of major skeletal muscle groups, initially focusing on the back muscles, followed by their antagonists—the chest muscles, incorporating synergistic muscles such as biceps, triceps, and shoulder muscles. The training concluded with squats, primarily engaging the lower limbs and gluteal muscles. Abdominal muscles, especially the transverse muscle, were engaged during the exercises to prevent lumbar spine injuries.

In the second week of intervention, after the body adapted to the training, the regimen transitioned to 3 sets of 6 exercises, with 90-second breaks between them. From the third week of intervention, the training comprised 3 sets of 9 exercises, with 60-second breaks between them.

The exercise order in the "push" set allowed for the initial engagement of chest muscles (barbell bench press, supported push-ups), followed by shoulder muscles (standing dumbbell press), triceps muscles (cable triceps extension), concluding the training by involving lower limb muscles through squats (supported sit-ups).

The "pull" set commenced with the engagement of back muscles in the first 3 exercises (one-arm row with dumbbell, reverse grip lat pulldown, bent dumbbell row). Subsequently, isolated exercises allowed for the engagement of biceps (standing dumbbell curl), hamstring muscles (dumbbell deadlift, hip thrust lying), concluding with the involvement of abdominal muscles (plank).

The participants who were examined underwent the 1 RM test prior to the assessment, as well as after 6, 12, and 16 weeks. A personal coach conducted a warm-up session on the Technogym New Excite Run Now 500 treadmill from Cesena, Italy, for 5 minutes at 60% of the heart rate. The individuals warmed up in two sets of 10 repetitions using approximately 50% of their estimated 1 RM load before starting the test protocol. Following a 5-minute break, the subjects were instructed to perform the designated test exercise until they were unable to continue the exercise series while maintaining proper technique (reaching failure). During the 1RM bench press test, the subjects were instructed to maintain contact between five points of their body (i.e., head, back, hips with the bench, and both feet with the floor) throughout the test. The barbell had to touch the chest when being lowered. In the 1RM squat test, the participants were instructed to move from a standing position to a position of 90 degrees of knee joint flexion. The pull-down test was conducted using a training atlas. Each repetition was considered valid when the subject fully extended their arms during the eccentric phase and touched the bar to their chest during the concentric phase. A qualified personal coach monitored the range of motion to ensure the accuracy of the test. The last repetition of each set was performed when the participant could no longer continue the exercise while maintaining proper technique. The obtained load and number of repetitions were converted to 1 RM values using the 1 RM calculator, applying the Brzycki formula [45].

References:

45. Grgic, J., Lazinica, B., Schoenfeld, B.J. & Pedisic, Z. Test-Retest Reliability of the One-Repetition Maximum (1RM) Strength Assessment: A Systematic Review. Sport. Med. Open. **6**, 31. DOI: [https://doi.org/10.1186/s40798-020-00260-z](about:blank) (2020).
